# Automated identification of copepods using digital image processing and artificial neural network

**DOI:** 10.1186/1471-2105-16-S18-S4

**Published:** 2015-12-09

**Authors:** Lee Kien Leow, Li-Lee Chew, Ving Ching Chong, Sarinder Kaur Dhillon

**Affiliations:** 1 Institute of Biological Sciences, Faculty of Science, University of Malaya, Kuala Lumpur, Malaysia; 2 Institute of Ocean & Earth Sciences, University of Malaya, Kuala Lumpur, Malaysia; 3 Center for Tropical Biodiversity Research, University of Malaya, Kuala Lumpur, Malaysia

**Keywords:** automated image recognition, copepods, Artificial Neural Network, digital image processing

## Abstract

**Background:**

Copepods are planktonic organisms that play a major role in the marine food chain. Studying the community structure and abundance of copepods in relation to the environment is essential to evaluate their contribution to mangrove trophodynamics and coastal fisheries. The routine identification of copepods can be very technical, requiring taxonomic expertise, experience and much effort which can be very time-consuming. Hence, there is an urgent need to introduce novel methods and approaches to automate identification and classification of copepod specimens. This study aims to apply digital image processing and machine learning methods to build an automated identification and classification technique.

**Results:**

We developed an automated technique to extract morphological features of copepods' specimen from captured images using digital image processing techniques. An Artificial Neural Network (ANN) was used to classify the copepod specimens from species *Acartia spinicauda, Bestiolina similis, Oithona aruensis, Oithona dissimilis, Oithona simplex, Parvocalanus crassirostris, Tortanus barbatus *and *Tortanus forcipatus *based on the extracted features. 60% of the dataset was used for a two-layer feed-forward network training and the remaining 40% was used as testing dataset for system evaluation. Our approach demonstrated an overall classification accuracy of 93.13% (100% for *A. spinicauda, B. similis *and *O. aruensis*, 95% for *T. barbatus*, 90% for *O. dissimilis *and *P. crassirostris*, 85% for *O. similis *and *T. forcipatus*).

**Conclusions:**

The methods presented in this study enable fast classification of copepods to the species level. Future studies should include more classes in the model, improving the selection of features, and reducing the time to capture the copepod images.

## Background

Copepods are the largest and most diversified group of crustaceans [1]. They are ubiquitous and the most abundant aquatic metazoans. Ecologically, copepods act as the most important link between phytoplankton and higher trophic levels in aquatic food webs. Copepods are sensitive to environmental disturbance and they can be the bioindicator for the changes in water quality [2]. Community shifts of copepods also provide sensitive indicator of climate change on marine biotopes [3]. Thus, copepods are one of the most studied microorganisms in marine food webs and fisheries studies. The size of adult copepods ranged from 200 µm to 2 mm in size, while their numbers can range up to 60,000 individuals per m^3 ^of water [4]. Positive identification of these organisms and completion of the work are thus hampered by their small size (mostly <0.20 mm in total length) and sheer numbers.

The identification of copepod species requires information of their morphology. Body shape is useful to characterise the genera, but may not be useful to differentiate closely related species. At the species and finer level, the characters of specific appendages such as the fifth legs are required [5]. Body shape and characteristics may however be useful to predict species in specific locations or habitats where the species are known or are low in diversity. Nevertheless, image capturing and processing tools for rapid and objective digital recognition of copepods at the familial or ordinal level are useful to non-specialists and ecologists.

Existing techniques in real time plankton-imaging-system are adequate for class/order-discriminations of plankton into major components [6]. One of the established studies known as ZOOSCAN digital imaging system described the zooplankton image processing and the semi-automatic recognition system using various machine learning methods [7]. In this semi-automatic recognition system, copepods were only covered in a few categories from the entire zooplankton community [8]. Hitherto, identification systems for calanoid copepods have been described in a few studies by using diffraction patterns as a tool [9-11,12] and the application of circular-harmonic filters [13].

Several classification methods such as neural network, structural, fuzzy and transform based techniques have been used in biological image identification systems but have not been employed for copepod classification. Artificial Neural Networks (ANN) [14] have shown satisfying results in complex classifications of biological images such as insects [15], microinvertebrates [16], algae [17,18], fish [19,20], leaves [19], butterflies [19], protozoans and metazoans [21], dinoflagellates [22] and human helminth eggs [23]. An ANN is a mathematical model composed of many processing units that communicate by interconnected variables [24]. Multilayer structure of ANN enables learning from complex input image features and generates single output [25].

This study aims to automate identification techniques to ultimately classify marine copepods down to the lowest or species level using image processing techniques to extract shape descriptors as features and the ANN algorithm as the classification tool. This approach is novel in copepods identification as previous studies only reported classification using diffraction pattern [9-12] and circular harmonic filter [13].

## Methods

**Figure 1:**
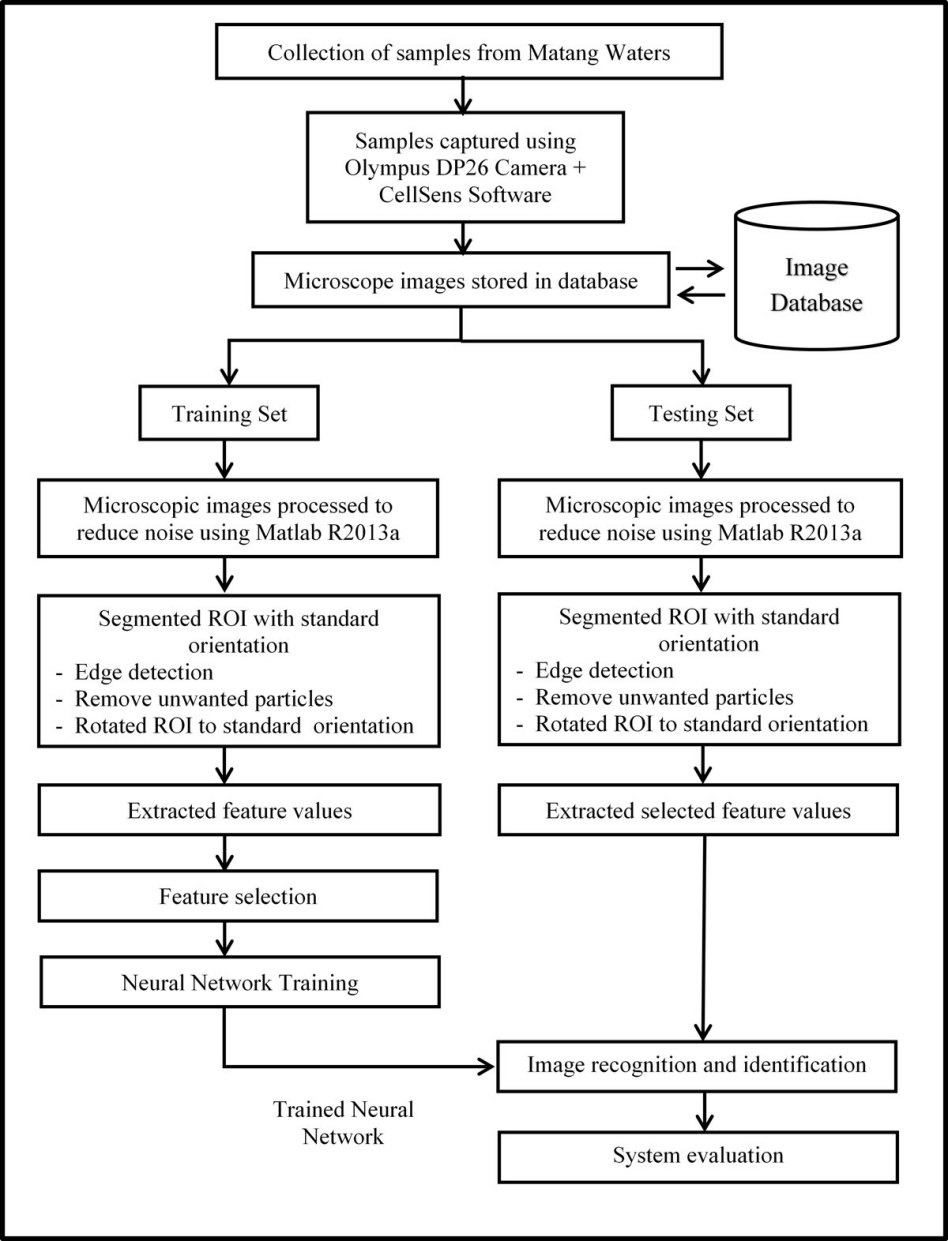
**System flowchart diagram**.

The study's approach followed the methodology and system flowchart illustrated in Figure 1 which are detailed as follows.

### Data collection

**Figure 2:**
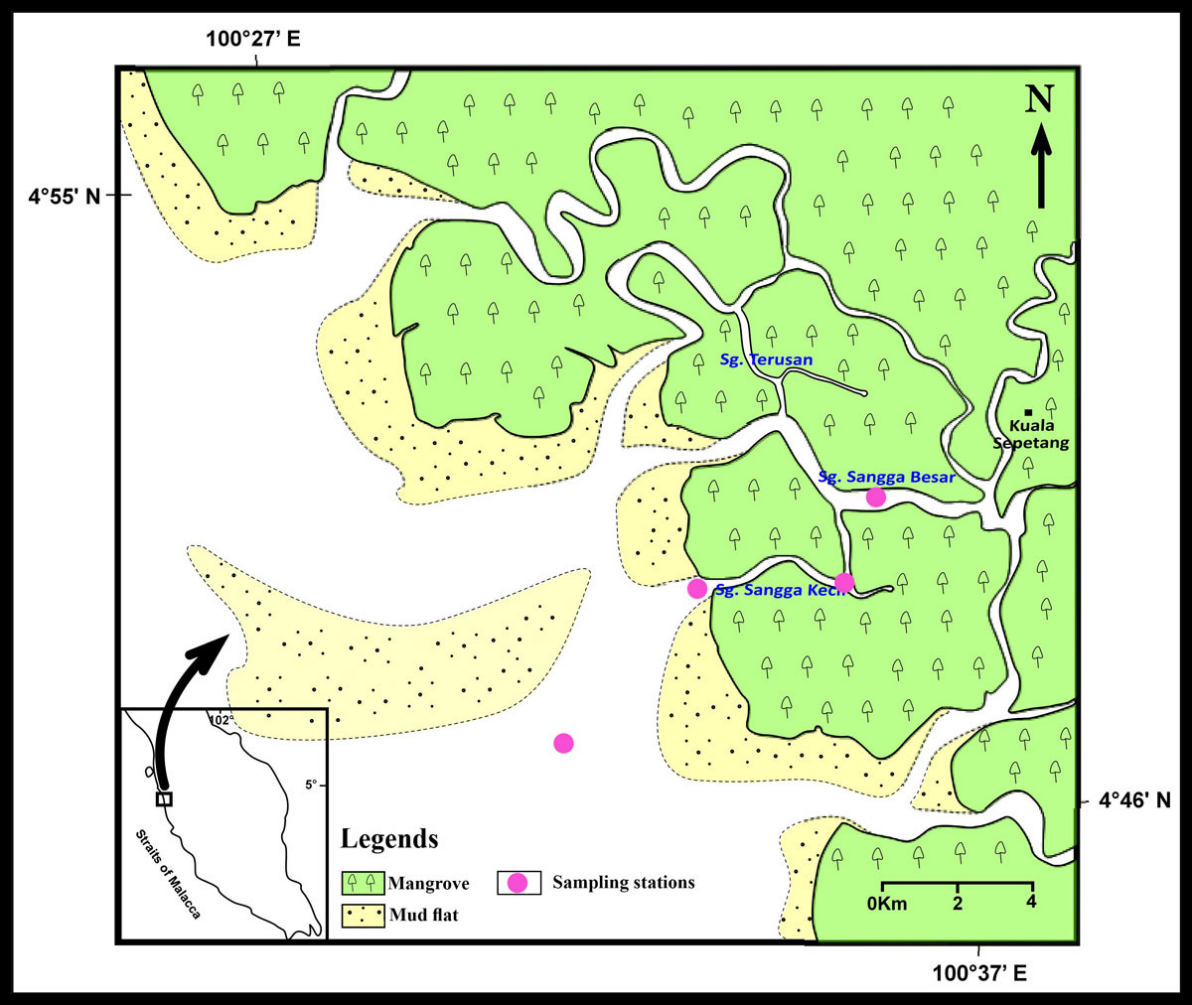
**Location of the sampling stations at Matang Mangrove Forest Reserve (MMFR)**.

Five genera of marine copepods commonly encountered in mangrove waters were examined: *Acartia *(*A. spinicauda*), *Bestiolina *(*B. similis*), *Oithona *(*O. aruensis, O. dissimilis *and O*. simplex*), *Parvocalanus *(*P. crassirostris*) and *Tortanus *(*T. barbatus *and *T. forcipatus*) (Additional File 1). Copepods were sampled from four stations from the upper estuary in the Matang Mangrove Forest Reserve (MMFR) to near shore waters on the west coast of Peninsular Malaysia (4°50'N, 100°35'E) (Figure 2). Horizontal plankton tows (0.5-1 m depth) using paired 45 cm-diameter bongo nets (180 µm) were made and collected plankton were preserved in buffered 10% formaldehyde. In the laboratory, collected copepods were then sieved through stacked Endecott sieves of 1,000 µm, 500 µm, 250 µm and 125 µm mesh sizes, and the sieved fractions were preserved in 80% alcohol in individual vials for a long-term preservation.

### Image acquisition

**Figure 3:**
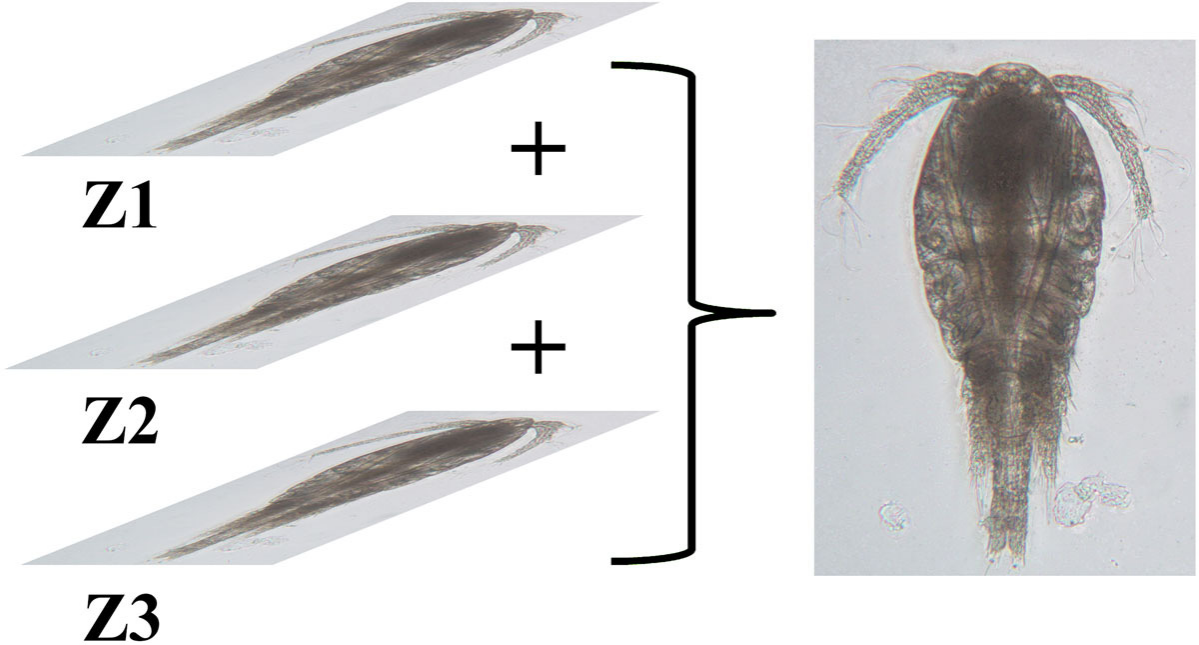
**Extended Focus Imaging (EFI)**. Image data at different focus was recorded to produce a single plane image.

**Figure 4:**
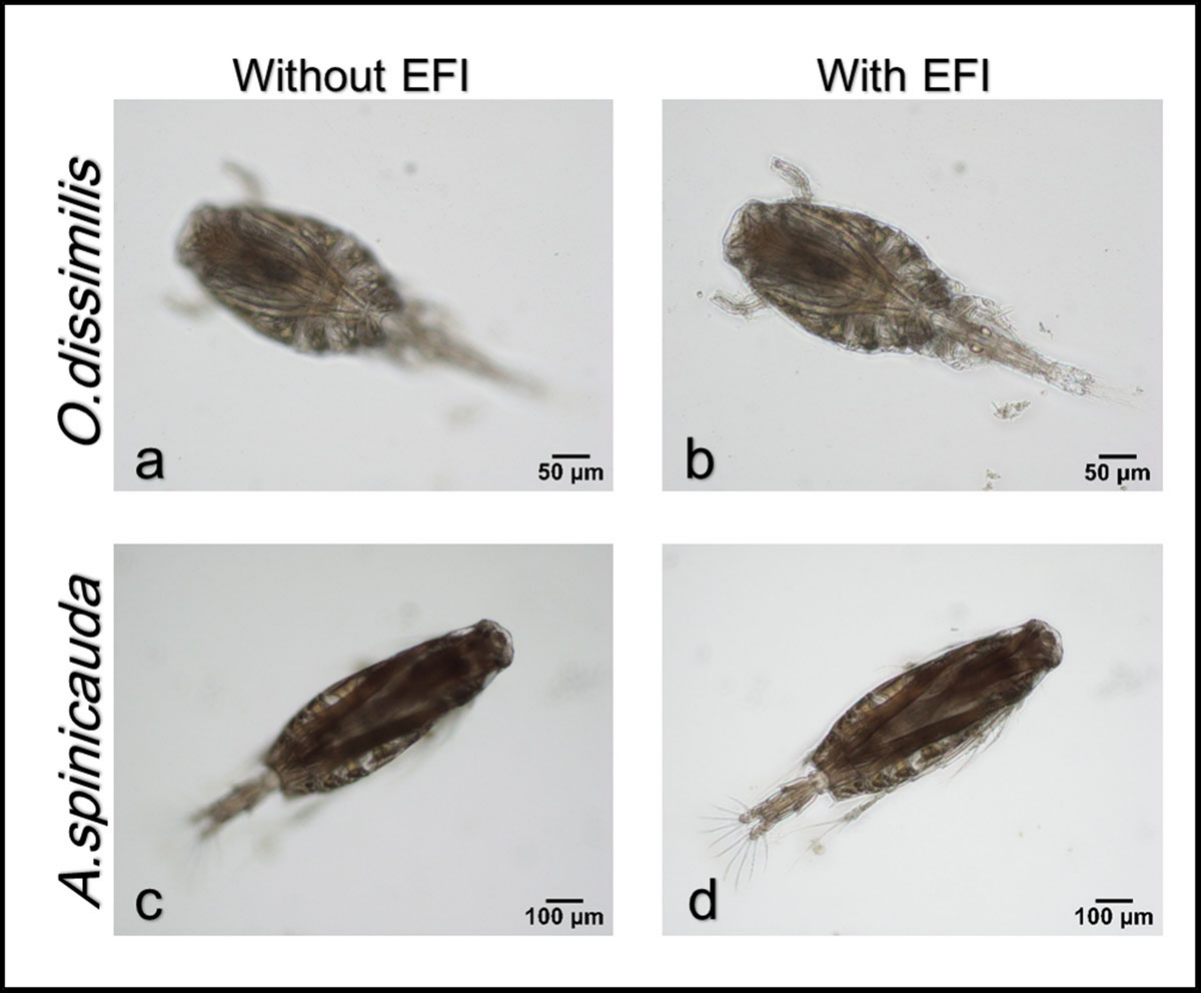
**Without EFI vs With EFI**. Comparison between captured images of ***O. dissimilis ***and ***A. spinicauda ***taken without (a and c) and with (b and d) EFI function.

Specimens of copepod were randomly pipetted onto a microscope slide from the preserved samples and each identified to species level under a compound microscope (Olympus BH2). To enable the dorsal aspect of the identified copepod to be imaged, often the copepod body had to be rotated. Body rotation could be easily achieved by first placing two short nylon fishing lines (0.36 mm diameter) on either side of the specimen and gently moving a cover slip placed over them by using the tip of the index finger. The desired view of the copepod body was imaged by an Olympus digital camera (DP26) connected to a computer installed with an imaging software (Olympus cellSens Standard ver. 1.12) [26] for real-time viewing, capturing and storing of the images. The built-in function in cellSens called Extended Focus Imaging (EFI) was used to create a single plane image with sharp, in-focus details and high contrast (Figure 3). The EFI function recorded the image data as the sample was gradually focused through from top to bottom to obtain single dorsal image of the copepod with all body parts (Figure 4). Besides, the contrast and brightness of the images were set to the best before they were captured using cellSens software. The resolution of the captured images was standardised (2448 × 1920 pixels) and all the images were saved in uncompressed Tagged Image File Format (TIFF) by renaming them according to the date when the images were captured.

### Image database

A simple image database was established to store and organise the captured images. Upon verification by copepod experts, these images were indexed according to their taxa. Thirty images for each species were stored as training set whereas twenty images of each species were stored as testing set.

### Image processing

Image processing was done in three essential steps: image pre-processing, image segmentation and feature extraction. The Image Processing Toolbox in Matlab R2013a [27] was installed on Intel(R) Xeon (R) CPU E5345 @ 2.33GHz, 4.00GB RAM, Windows 7 Professional (32-bit) to conduct this study.

**Figure 5:**
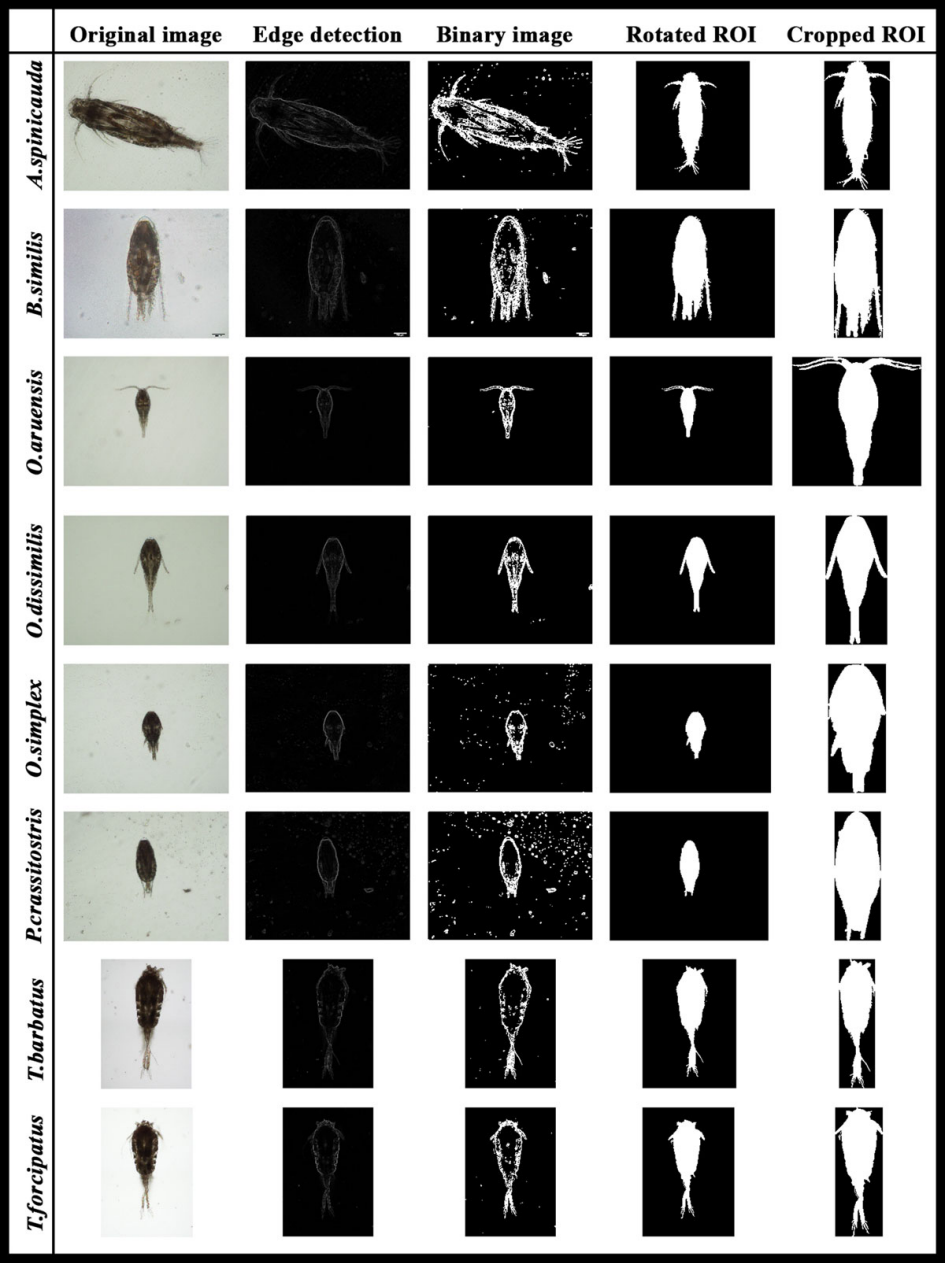
**Process in image pre-processing, edge detection and image segmentation steps**.

The captured images were pre-processed in the following steps (Figure 5):

1) The images were first converted to 2-dimensional grayscale images.

2) A median filtering with a 10-by-10 kernel was used to suppress the noise found in the images which mainly consisted of salt-and-pepper noise from the water.

3) A 2-D order-statistic filtering algorithm with 10-by-10 domain was applied to detect the edge of the copepods. In this basic gradient-based segmentation function, the edge was derived from the difference between the first (*ordfilt2(1)*) and the last order-statistic filter (*ordfilt2(100)*).

**Figure 6:**
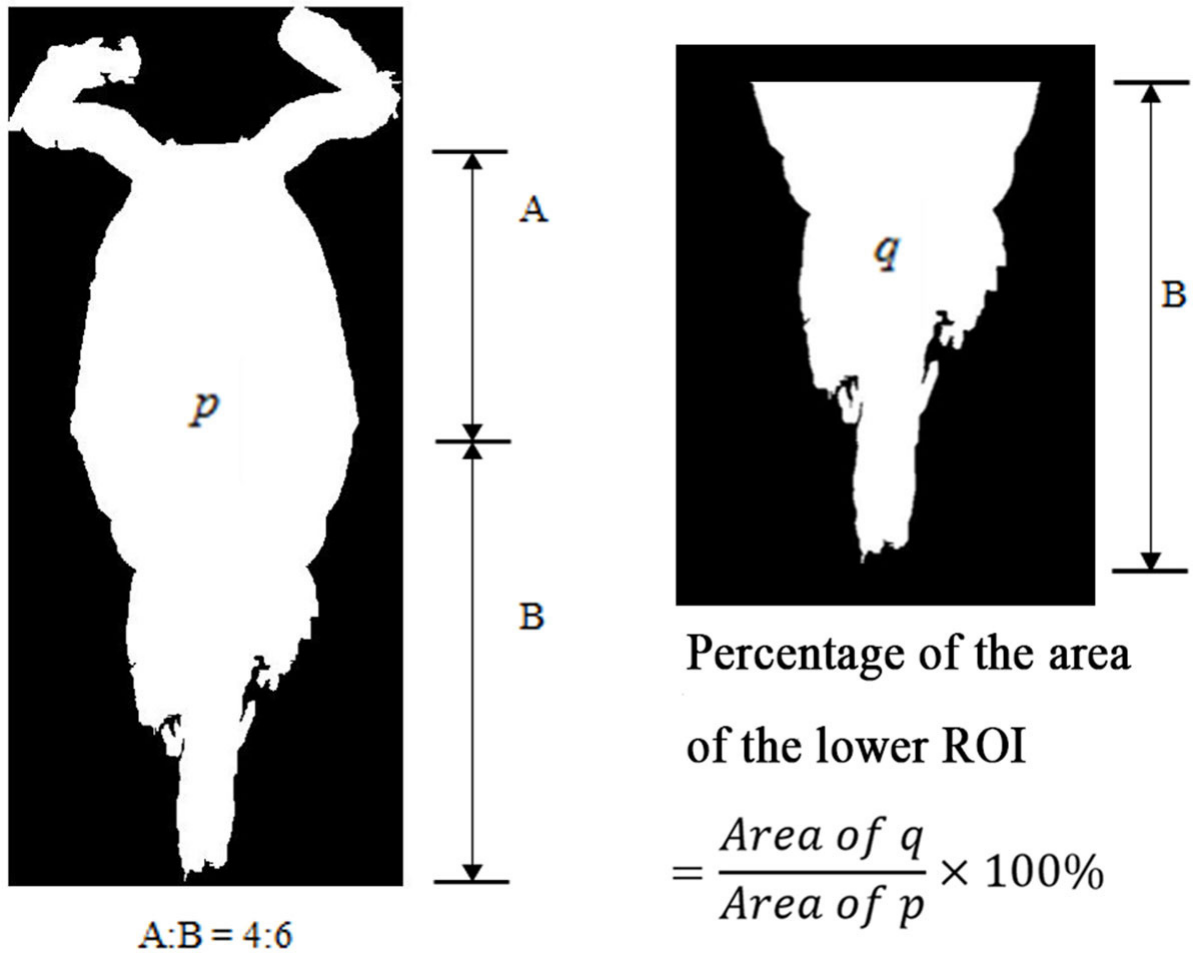
**Percentage of area of the lower ROI image**.

Once the edges in the images were detected, the following steps were then taken for image segmentation where copepods were identified and segmented from unwanted particles in the images:

1) The images were converted to binary images with appropriate threshold.

2) The borders in the images were cleared using the *imclearborder *function and the holes that occurred during the process of converting the grayscale image into binary image were filled using the *imfill *function.

3) Small particles (<50000 pixels) were excluded to ensure only the copepods are segmented for feature extraction.

4) The orientation represented by the angle between the x-axis and the major axis of the ellipse that has the same second-moments as the region of interest (ROI) was obtained using region properties function in Matlab. Image rotation was done using the *imrotate *function so that the ROI has an orientation of 90 degrees.

5) The ROI of the copepod was cropped by getting the coordinates of the boundary of copepods.

6) Features were extracted from the shape descriptors represented by the binary images of the ROI using region properties function in Matlab. The measurements taken were area, convex area, eccentricity, major axis length, minor axis length, perimeter, solidity, equivdiameter (sqrt(4*area/pi)), extent and orientation.

7) As seen in the ROI images of copepod, the lower part showed distinct shapes across the eight species. In view of this distinct attribute, a secondary feature was derived by assigning 60% of the ROI image height measured from the posterior end (end of urosome) to the anterior end (head of copepod) of copepod body as the lower part of ROI image. This ratio was selected after conducting several tests using a set of ratios (90%, 80%, 70%, 60% and 50%). This derived feature was calculated as: (Figure 6)

Percentage of area of the lower part of ROI image

$$=\frac{Area\;of\;q}{Area\;of\;p}\times 100\%$$

Where *p *is the total area of ROI image and *q *is the area of the lower part of ROI image.

### Feature selection

**Figure 7:**
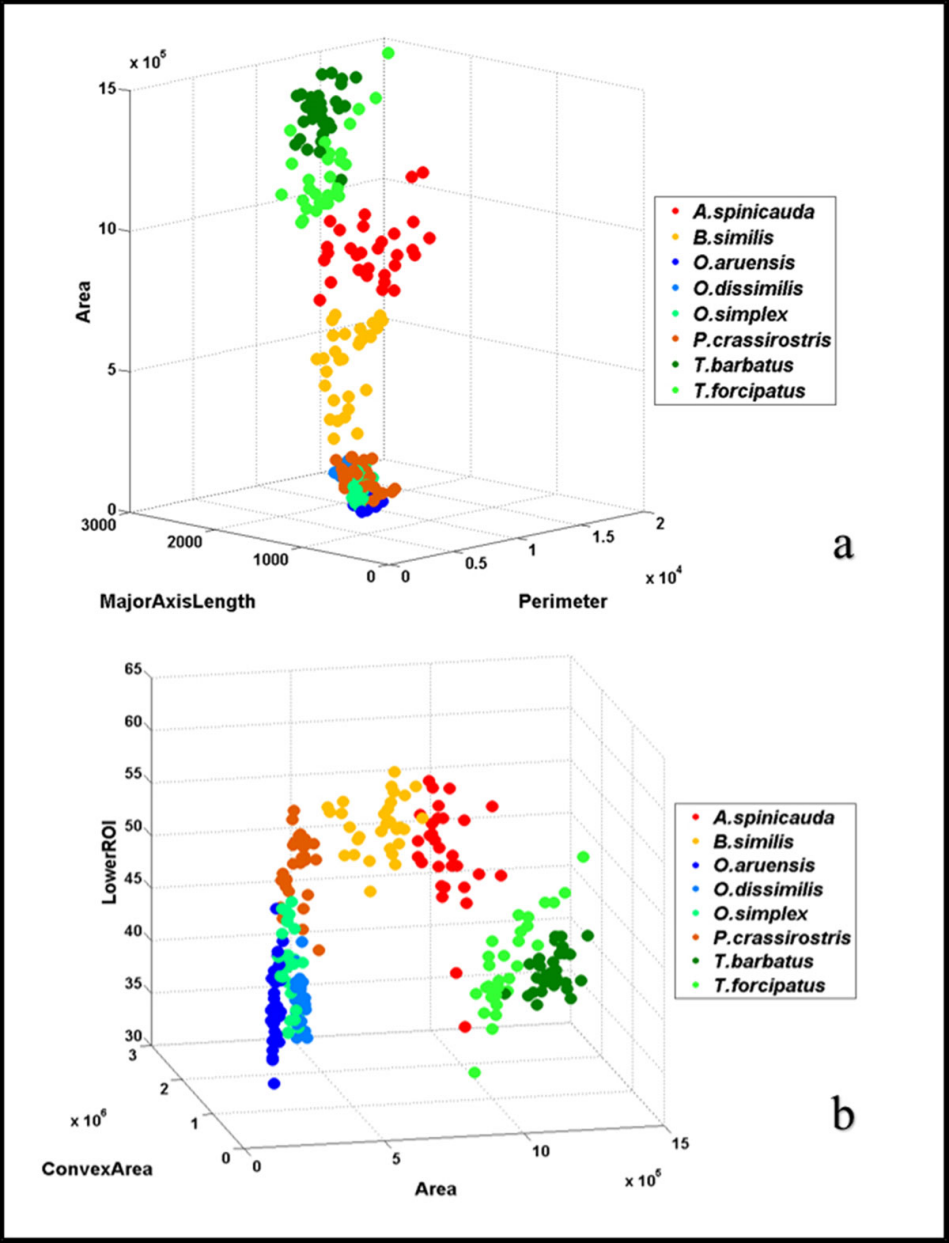
**3D scatter plots with different combinations of features**. (a) Area vs. MajorAxisLength vs. Perimeter; and (b) LowerROI vs. ConvexArea vs. Area are two different combinations of features used to select the features for classification. Features that are able to group specimens into eight distinct groups were chosen for neural network training.

To avoid overfitting in the Neural Network training and to increase performance, not all the 11 extracted features were used. The extracted features were evaluated to make sure that only significant features were selected to classify the copepods into their respective taxa. Forward stepwise discriminant analysis (FSDA) was used to aid the selection of the most useful features (StatSoft Inc.). In order to visualise how well a selected feature clustered the specimens in the training set into the eight classes (species), 2D and 3D scatter plots were graphed (see Figure 7) with different combinations of features as the axes.

### Neural Network training

**Figure 8:**
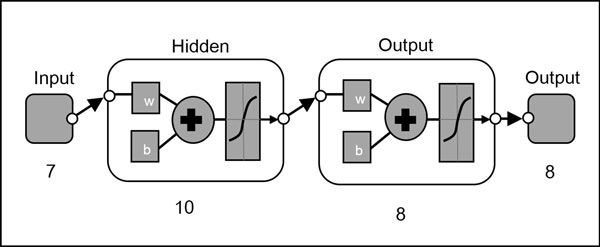
**Pattern recognition neural network diagram**.

An Artificial Neural Network (ANN) was used as the pattern recognition tool to classify the extracted features values into the eight classes (species). The architecture of the ANN is a two-layer feed-forward network with sigmoid hidden (ten nodes) and output (eight nodes) neurons and the network was trained with scaled conjugate gradient backpropagation (Figure 8). A total of 240 sample images were used in the training set with 30 images from each class. The input data presented to the input nodes of the network contained seven selected features of each specimen from the training set, whereas the target data defined eight desired output classes. The 240 samples were then divided into three sets, the training set (168 samples, or 70% of samples), validation set (36 samples, 15%) and testing set (36 samples, 15%). The data from the training set were used for network training; the validation set for measuring network generalisation and terminating training before overfitting; and the testing set for independent measure of network performance during and after training. The performance of the network training was evaluated using Mean Square Error (MSE) and confusion matrices. The training stopped when the MSE of the samples in the validation set started to increase indicating that the network generalisation stopped improving. The network was trained several times to get the trained network with best performance. Another 160 independent samples (20 samples for each species) were used for system performance evaluation. The trained network was simulated using the testing data as input and the output was then compared to the predicted data and recorded in a confusion matrix.

## Results

### Feature selection

**Figure 9:**
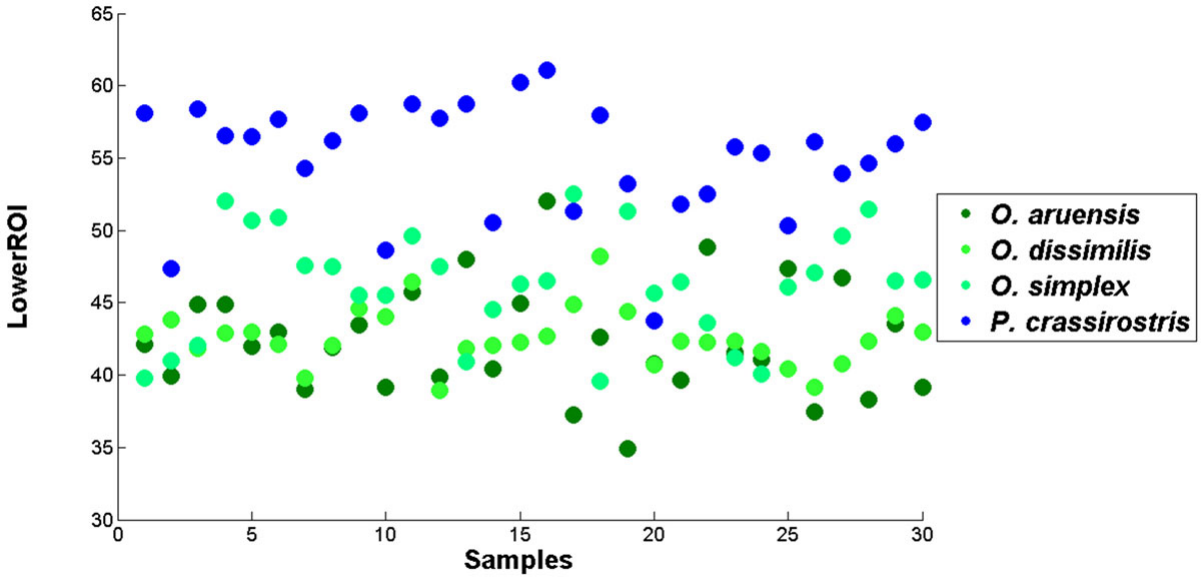
**2D scatter plot for percentage of area of the lower ROI image for samples from genus *Oithona *and *Parvocalanus***. The plot shows that this feature managed to separate samples from these two genera that are very similar.

A total of 11 copepod features were initially extracted from the samples but only seven of them were finally chosen to avoid overfitting in the neural network training. The seven selected features were area, convex area, major axis length, minor axis length, perimeter, equivalent diameter and percentage of lower ROI image. Although FSDA by default settings selected 10 features (except "orientation") as significant in the classification model, the final seven features were selected based on the F-value associated with their partial Wilks' Lambda (i.e. those that contributed most to the discriminatory power of the model). These features when visualized on the 2D and 3D plots gave clusters of species with little overlaps (Figure 7). Interestingly, the secondary feature (lower ROI) is seen to separate genus *Oithona *from genus *Parvocalanus *(Figure 9).

### Neural Network training

**Figure 10:**
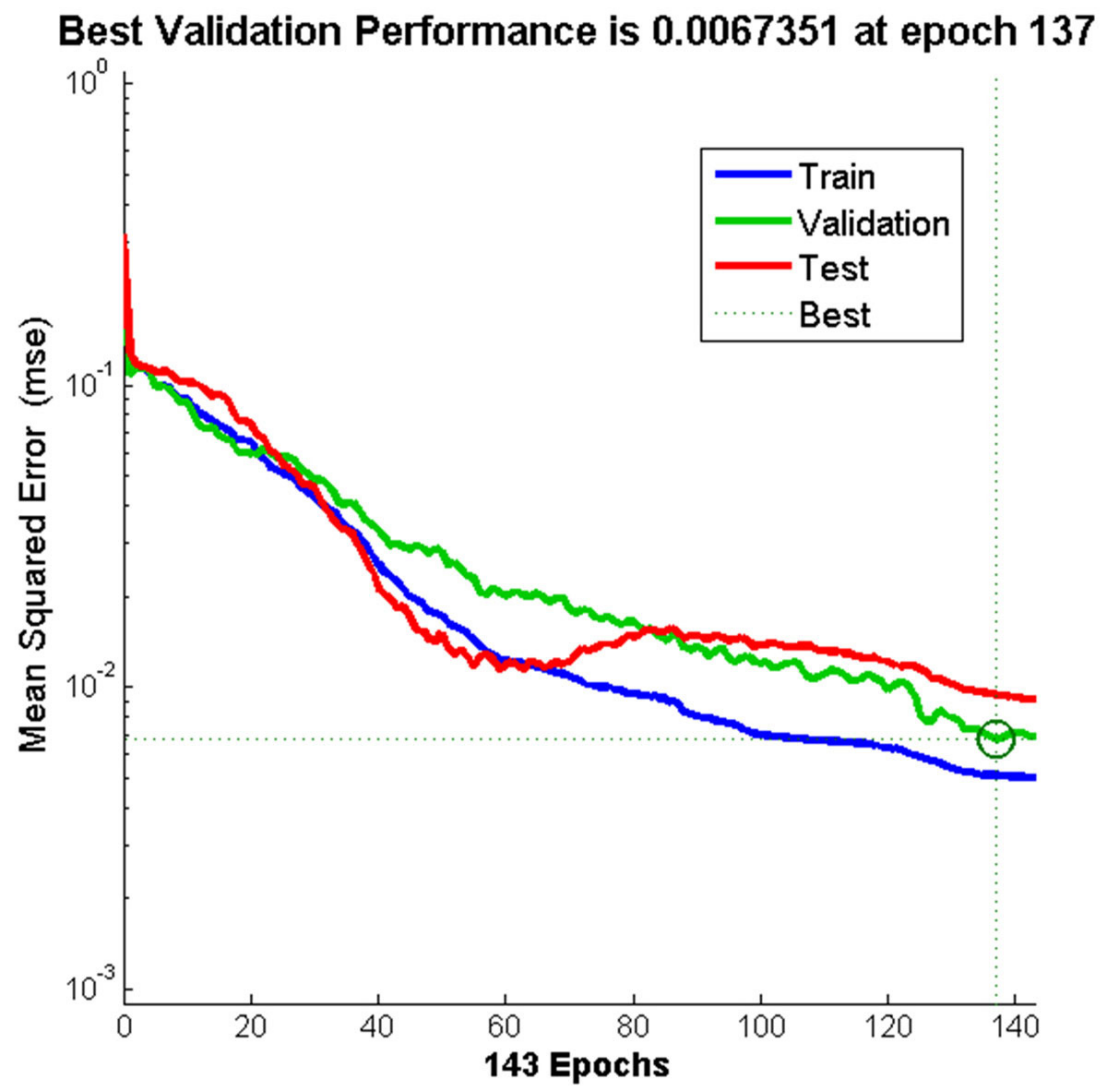
**Neural Network training performance**. The training stopped once the MSE for validation data started to increase at epoch 137.

**Figure 11:**
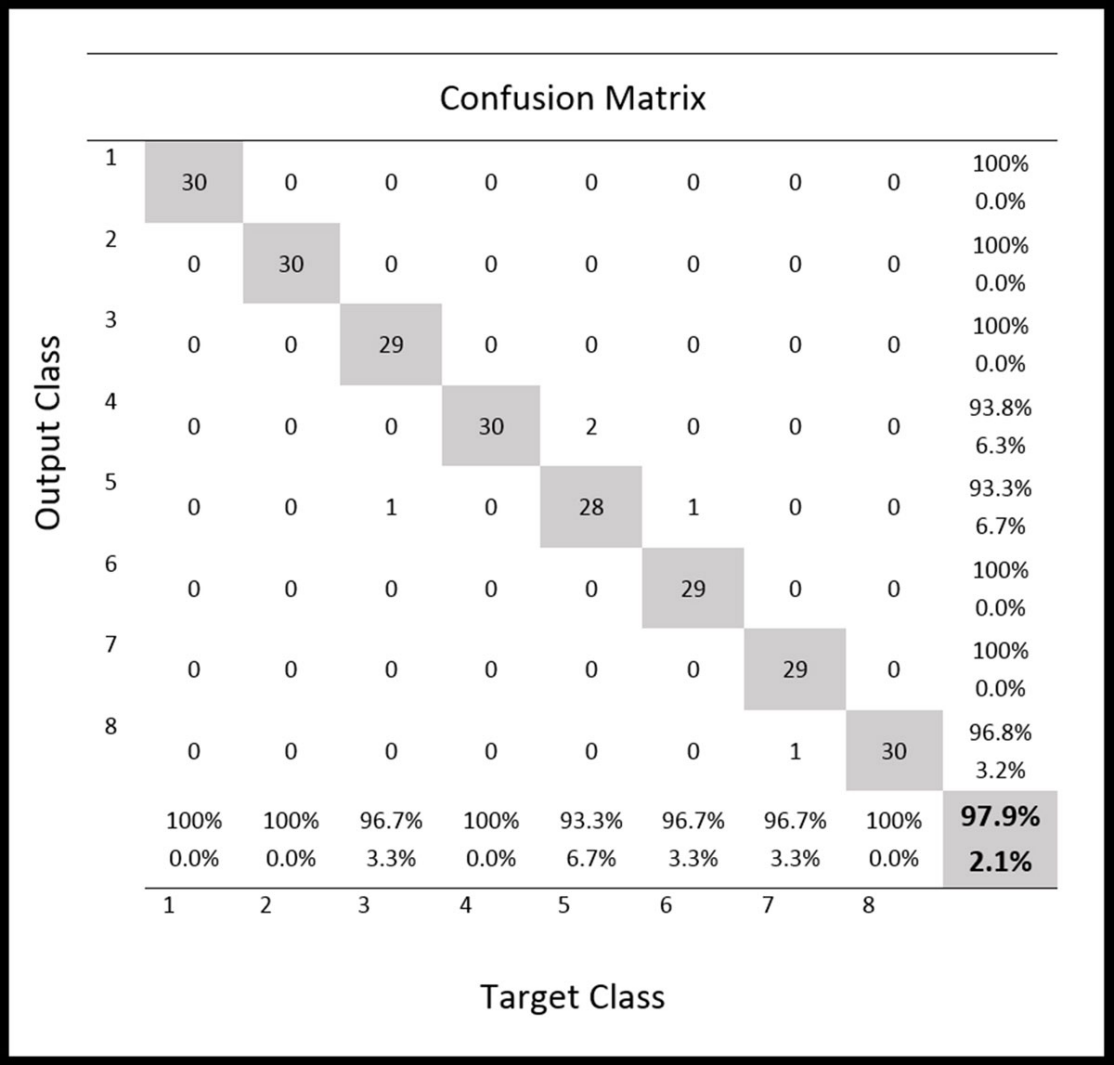
**Confusion matrix from network training**. Confusion matrix shows the classification of 8 species of marine copepods for training, validation and testing samples from the training of two-layer feed-forward network.

A two-layer feed-forward network was trained with back propagation algorithm based on ten neurons at the hidden layer and eight neurons at the output layer. The best trained network was obtained with 143 iterations. The best validation performance in the trained network had a MSE of 0.0067 at epoch 137 (Figure 10). Result from the confusion matrix showed overall 97.90% of correct classification of all 240 samples in the training, validation and testing sets (Figure 11).

### System evaluation

**Figure 12:**
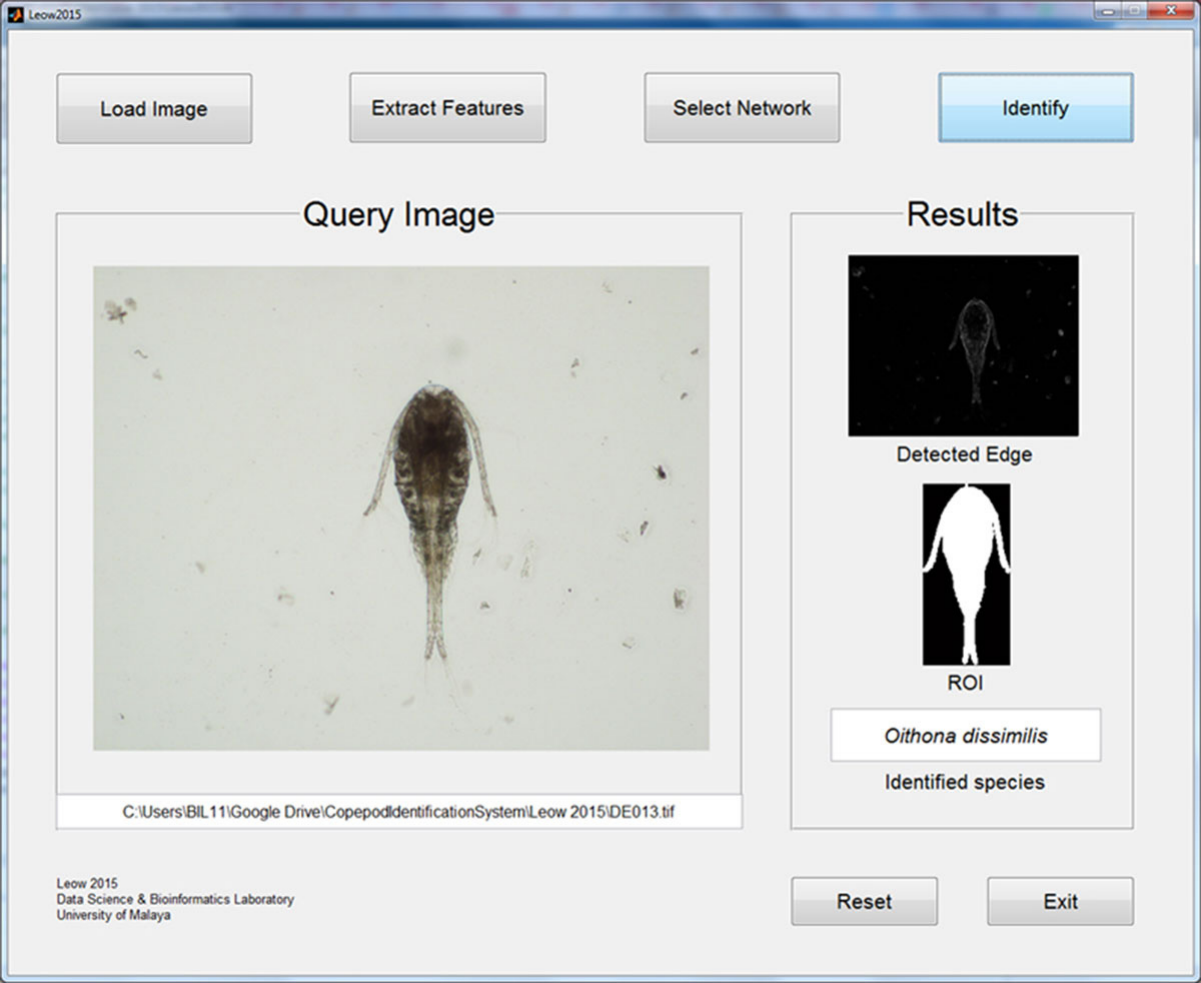
**Graphical User Interface (GUI) for automated identification of copepods**. Only four simple steps are needed for the system to identify a copepod species from an image.

A Graphical User Interface (GUI) was created for the automated identification system as shown in Figure 12. The GUI allows users to perform loading of input images, feature extraction, selection of network and species identification. The performance of the system was evaluated by comparing the output from the trained network to the identification result of the copepodologists using the testing dataset as the input. The testing dataset that was used to simulate the trained network was a new independent dataset not used for the network training. The results show that the technique presented in this study was capable of identifying most of the copepods correctly with an overall accuracy of 93.13% (Table 1). All *A. spinicauda, B. similis *and *O. aruensis *specimens were identified correctly; one specimen from *T. barbatus *and three specimens of *T. forcipatus *were misidentified as each other; two specimens from *O. dissimilis *was misidentified as *O. simplex*; two specimens from *P. crassirostris *were misidentified as *O. aruensis *and *O. simplex*; three specimens of *O. simplex *were misidentified as *O. dissimilis *and *P. crassirostris*. Another confusion matrix (Table 2) was prepared to show the classification result to genus level. An overall accuracy of 98.13% was achieved where only one specimen from *Oithona *and two specimens from *Parvocalanus *were misidentified as each other.

**Table 1:** Confusion matrix of testing dataset used for system evaluation (identification to species level).

Species	Results								Accuracy %
		
	*As*	*Bs*	*Oa*	*Od*	*Os*	*Pc*	*Tb*	*Tf*	
*As*	20	0	0	0	0	0	0	0	100
*Bs*	0	20	0	0	0	0	0	0	100
*Oa*	0	0	20	0	0	0	0	0	100
*Od*	0	0	0	18	2	0	0	0	90
*Os*	0	0	0	2	17	1	0	0	85
*Pc*	0	0	1	0	1	18	0	0	90
*Tb*	0	0	0	0	0	0	19	1	95
*Tf*	0	0	0	0	0	0	3	17	85

**Overall**									**93.13**

The data were classified into 8 species: *Acartia spinicauda *(*As*), *Bestiolina similis *(*Bs*), *Oithona aruensis *(*Oa*), *Oithona dissimilis *(*Od*), *Oithona simplex *(*Os*), *Parvocalanus crassirostris *(*Pc*), *Tortanus barbatus *(*Tb*) and *Tortanus forcipatus *(*Tf*).

**Table 2:** Confusion matrix of testing dataset used for system evaluation (identification to genus level).

	Results					
Genus						Accuracy %
	*Aca*	*Bes*	*Oit*	*Par*	*Tor*	
*Aca*	20	0	0	0	0	100
*Bes*	0	20	0	0	0	100
*Oit*	0	0	59	1	0	98.3
*Par*	0	0	2	18	0	90
*Tor*	0	0	0	0	40	100

**Overall**						**98.13**

The data were classified into five genera: *Acartia *(*Aca*), *Bestiolina *(*Bes*), *Oithona *(*Oit*), *Parvocalanus *(*Par*) *and Tortanus *(*Tor*).

## Discussion

The purpose of the study is to present an automated identification and classification technique for copepods based on the captured images to lighten and assist the work of non-specialists or ecologists. Extended focus imaging (EFI) technique was used to capture copepod images under the microscope using cellSens software to produce high quality images of copepods; in order to provide more information and features that could be extracted. The antennae of specimens *T. barbatus *and *T. forcipatus *were removed as rotation to the desired dorsal aspect could twist its bulky antennae to awkward positions resulting in some feature values to deviate; this may lead to misclassification. Although a desired dorsal-up orientation was required for image acquisition, this was not always perfect since the copepod body might tilt slightly. Hence, image rotation was performed to make sure the sagittal plane of the copepod was perpendicular to the horizontal axis of the image. All captured images were stored in a simple image database to ease the retrieval of particular images for network training and system evaluation. From the results, an overall accuracy of 93.13% was achieved for the testing set where the identification of *A. spinicauda, B. similis *and *O. aruensis *was 100% correct, while the identification of other species achieved 85% to 95% accuracy. *A. spinicauda, B. similis *and *O. aruensis *are distinct in terms of body size, shape and other features and are thus easily identified. *O. dissimilis *tend to be misidentified as *O. simplex *as they are from the same genus; same goes to *T. barbatus *and *T. forcipatus *from genus *Tortanus. O. simplex *and *P. crassirostris *tend to be misclassified as the other because they have similar sizes and other features despite the use of an additional feature (percentage of the lower ROI image) to differentiate them. In terms of classification at genus level, an accuracy of 98.13% was achieved showing an increase in accuracy compared to identification at species level. The seven features selected for neural network training produced an overall accuracy of 93.13%. Number of features for neural network training does not guarantee increase in overall performance. What matters most is the types of features selected. It is crucial to select only features that are able to cluster the specimens into distinct groups before the network training.

The present copepod identification technique used shape descriptors as distinguishing features and an ANN as the pattern recognition tool to identify and classify copepods. This technique differs from those used by previous workers, such as Zavala-Hamz et al. (1996), Castro-Longoria et al. (2001) and Alvarez-Borrego & Castro-Longoria (2003) who used correlation analysis of the diffraction pattern of digitised copepod images. In this study, the time taken for digitising the copepod images can be improved with the help of new technologies in plankton-imaging. Thus, development in hardware technology will determine the future prospects and application of automated identification systems in ecological studies. In the future, we plan to use more genera including more species. Besides, other aspects like gender and life cycle stages of copepods could be taken into consideration.

## Conclusions

The present technique of automated identification of copepods to species level based on dorsal images of copepods under the microscope achieved an overall accuracy of 93.13%. The approach used image processing technique to extract features from microscope images and an ANN as the classifier. Aquatic ecologists will find the automated identification method useful since samples processing time will be reduced and effort can be spent on other ecological related works. Future work should focus on the enhancement of image acquisition and feature extraction techniques to accommodate large datasets covering more taxa. Ultimately, the aim is to develop a fully automated identification system capable of identifying copepod specimens down to the lowest taxonomic level.

## Competing interests

The authors declare that they have no competing interests.

## Authors' contributions

SKD headed the study, structured the whole research and contributed to the writing of the manuscript. LLK collected specimens, designed and implemented the methods as his Msc. study and played a major role in manuscript writing. CLL assisted in sample collection and provided expert identification of copepods. CVC provided laboratory facilities and contributed to the writing of the manuscript. All authors contributed in this study. All authors read and approved the final manuscript.

## Supplementary Material

Additional file 1 **Sample images of copepods from eight species used in the study**. The eight species included ***A. spinicauda***, ***B. similis***, ***O. aruensis***, ***O. dissimilis***, ***O. simplex***, ***P. crassirostris***, ***T. barbatus ***and ***T. forcipatus***. Click here for file

## Declarations

Publication charges for this article was funded by University of Malaya Page Charge Fund and University of Malaya's Postgraduate Research Fund (PG130-2013A).

This article has been published as part of *BMC Bioinformatics *Volume 16 Supplement 18, 2015: Joint 26th Genome Informatics Workshop and 14th International Conference on Bioinformatics: Bioinformatics. The full contents of the supplement are available online at http://www.biomedcentral.com/bmcbioinformatics/supplements/16/S18.
