# Supplementary material for: Automated identification of copepods using digital image processing and artificial neural network
Source: BMC Bioinformatics. 2015 Dec 9;16(Suppl 18):S4. doi: 10.1186/1471-2105-16-S18-S4 (PMC4682403; doi:10.1186/1471-2105-16-S18-S4)

Additional file 1 –
Sample images of copepods from 8 species used in the study.

Acartia spinicauda
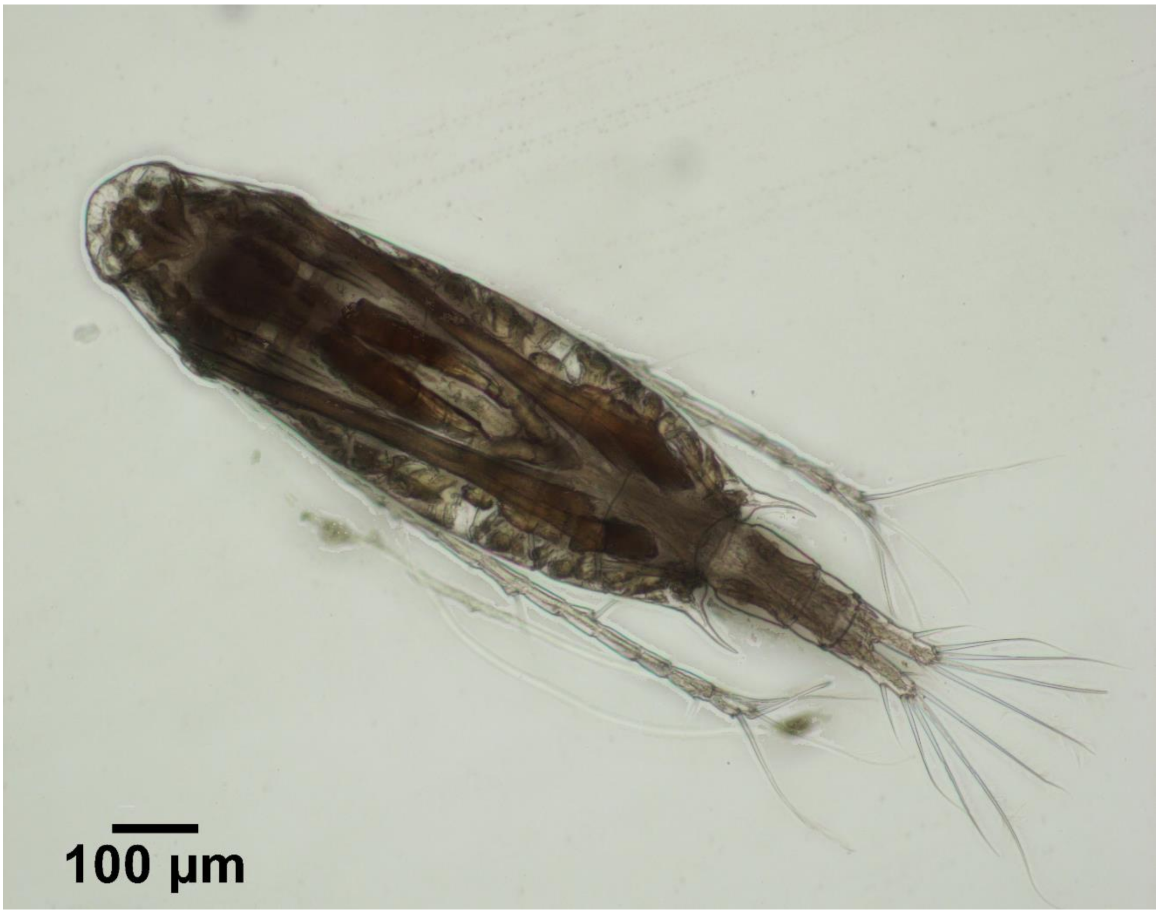

Bestiolina similis

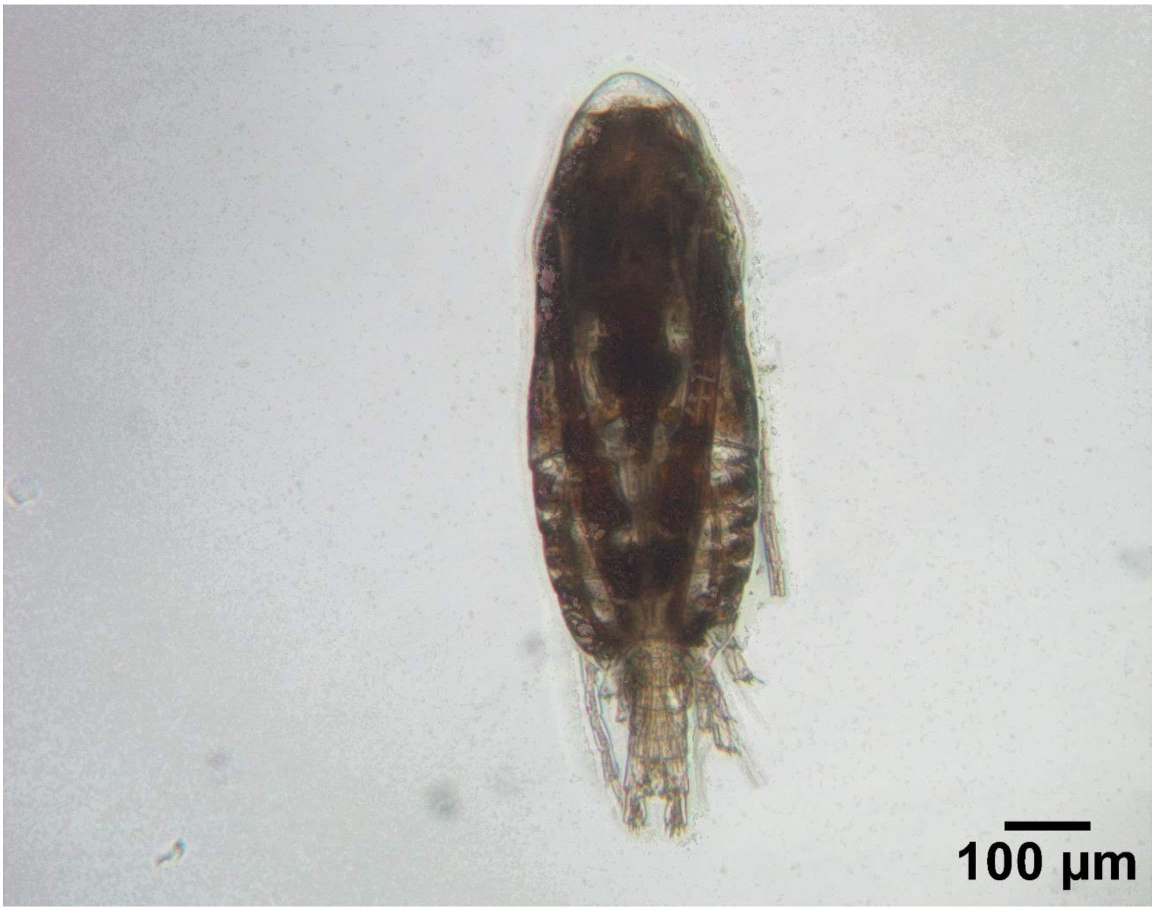

Oithona aruensis

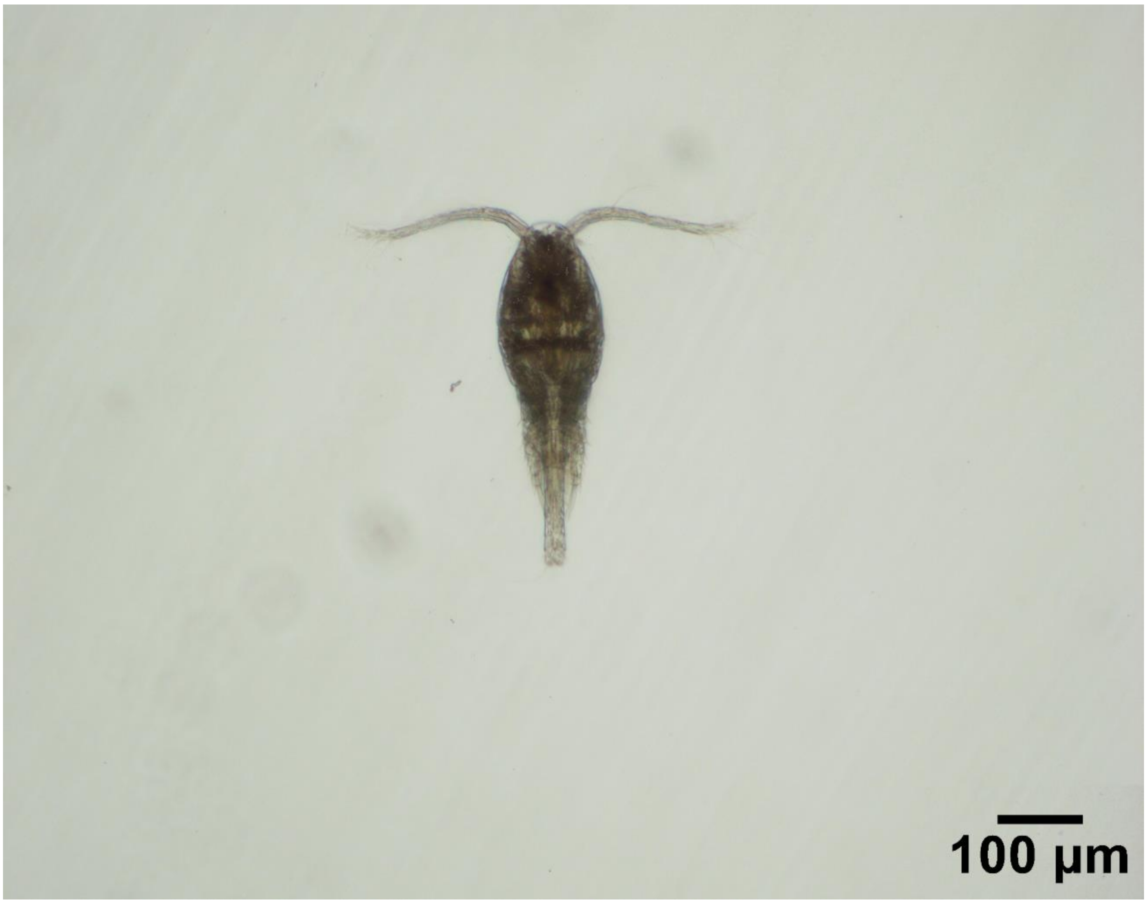

Oithona dissimilis
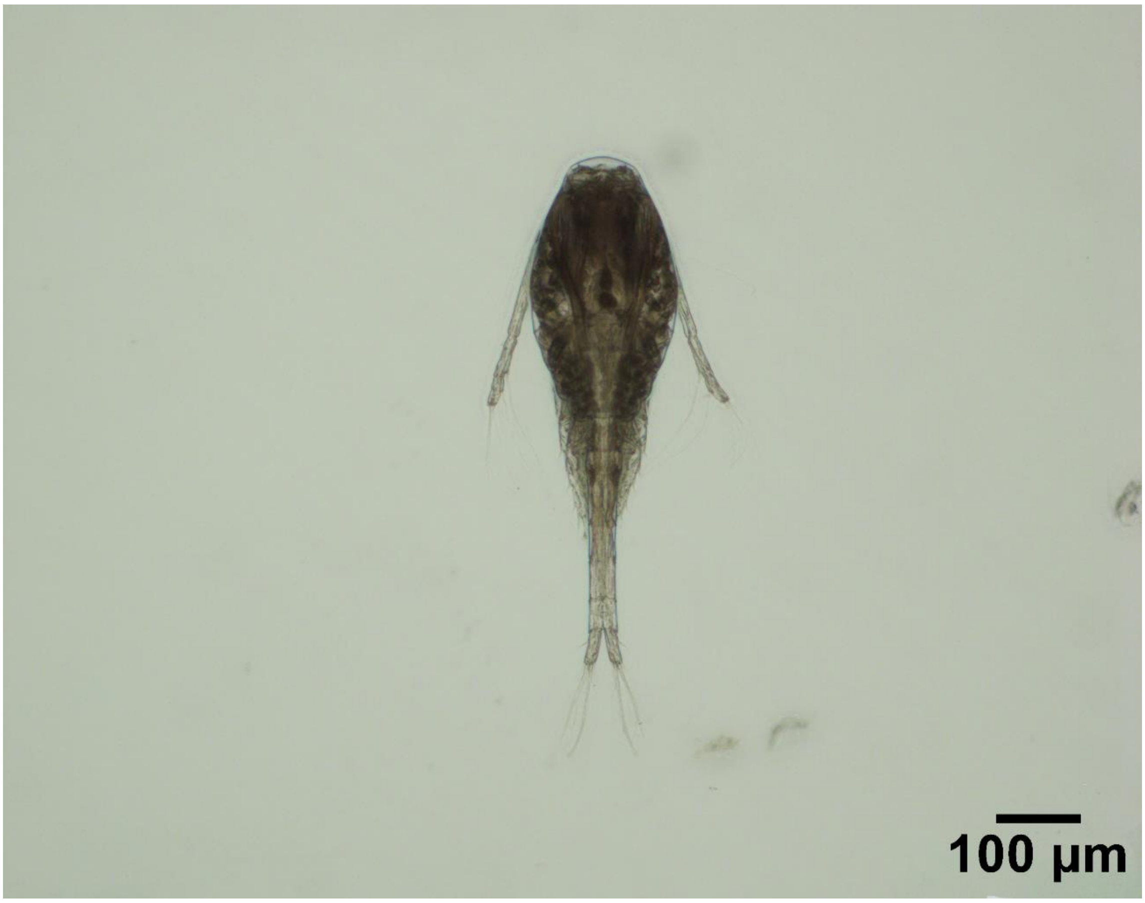

Oithona simplex
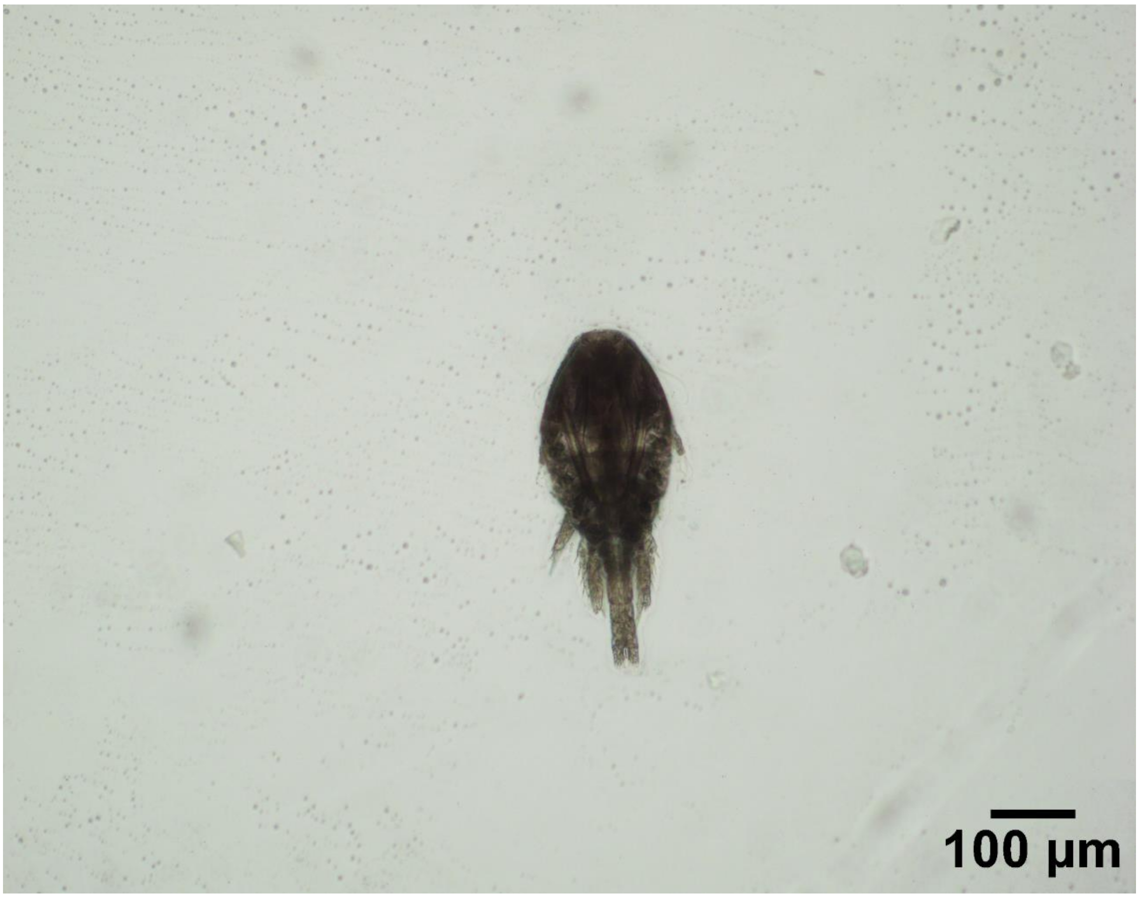

Parvocalanus crassirostris
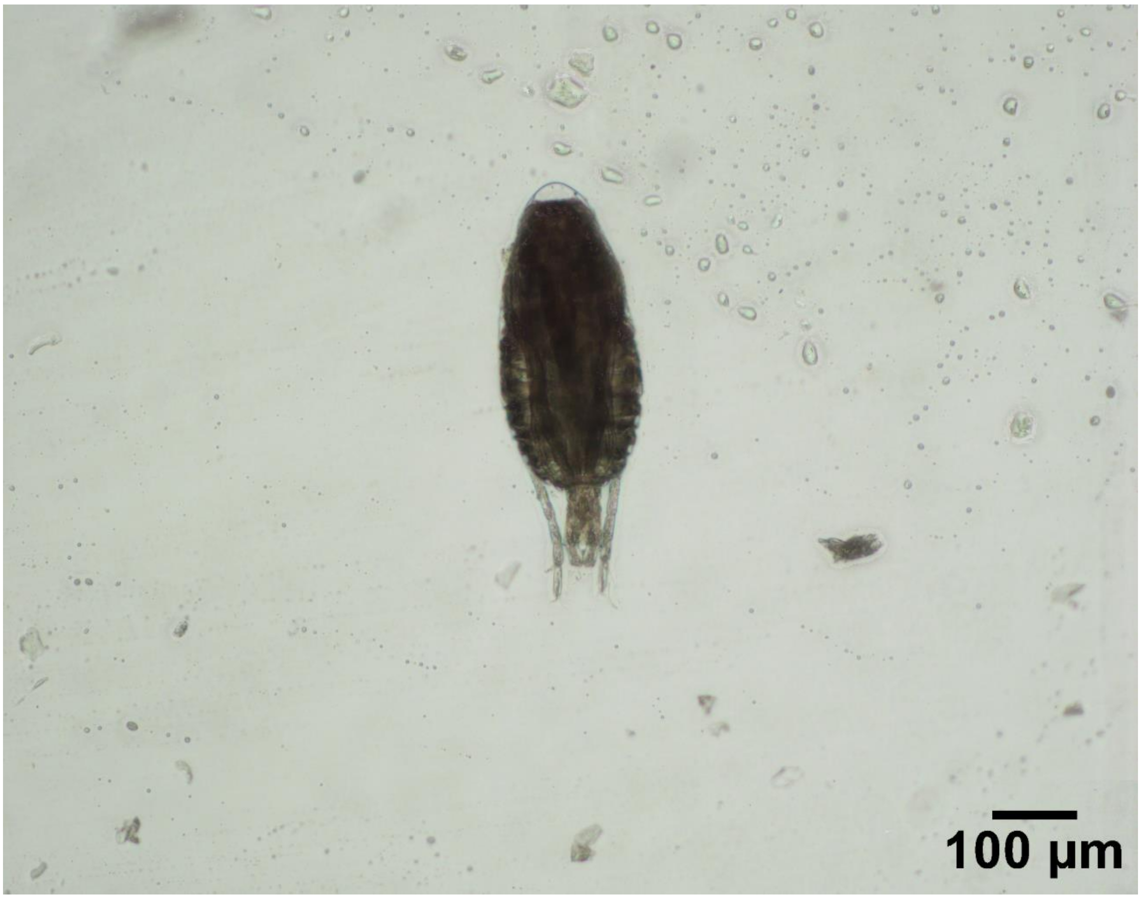

Tortanus barbatus

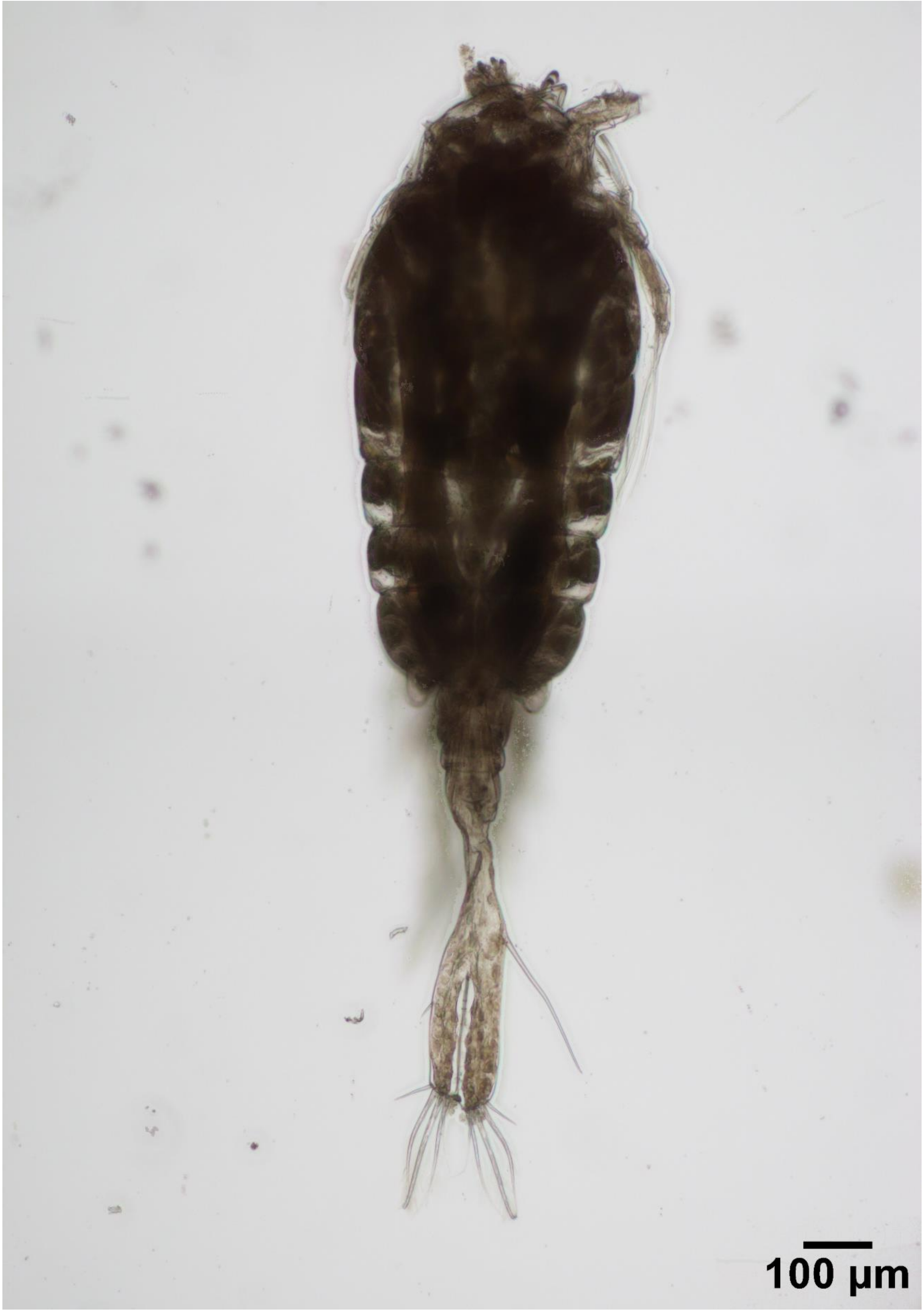

Tortanus forcipatus

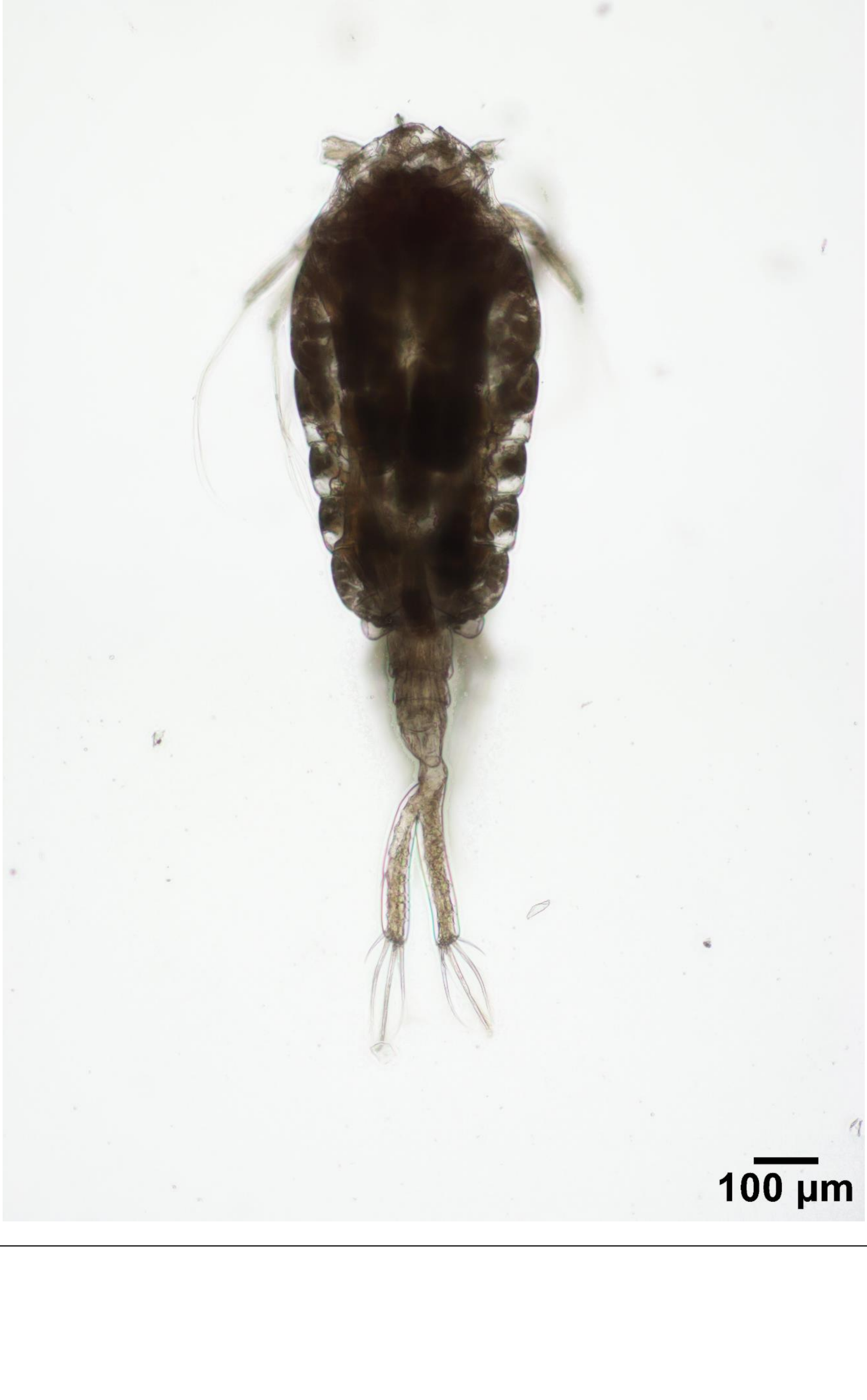

Supplement: Additional file 1 — Sample images of copepods from eight species used in the study. The eight species included A. spinicauda, B. similis, O. aruensis, O. dissimilis, O. simplex, P. crassirostris, T. barbatus and T. forcipatus. [file 1471-2105-16-S18-S4-S1.pdf]
